# Intermuscular Fat Content in Young Chinese Men With Newly Diagnosed Type 2 Diabetes: Based on MR mDIXON-Quant Quantitative Technique

**DOI:** 10.3389/fendo.2021.536018

**Published:** 2021-03-30

**Authors:** Fuyao Yu, Bing He, Li Chen, Fengzhe Wang, Haidong Zhu, Yanbin Dong, Shinong Pan

**Affiliations:** ^1^ Department of Radiology, Shengjing Hospital of China Medical University, Shenyang, China; ^2^ Department of Endocrinology, Shengjing Hospital of China Medical University, Shenyang, China; ^3^ Department of Medicine, Medical College of Georgia, Georgia Prevention Institute, Augusta, GA, United States

**Keywords:** type 2 diabetes, insulin resistance, skeletal muscle, intermuscular fat, MRI

## Abstract

**Objective:**

Skeletal muscle fat content is one of the important contributors to insulin resistance (IR), but its diagnostic value remains unknown, especially in the Chinese population. Therefore, we aimed to analyze differences in skeletal muscle fat content and various functional MRI parameters between diabetic patients and control subjects to evaluate the early indicators of diabetes. In addition, we aimed to investigate the associations among skeletal muscle fat content, magnetic resonance parameters of skeletal muscle function and IR in type 2 diabetic patients and control subjects.

**Methods:**

We enrolled 12 patients (age:29-38 years, BMI: 25-28 kg/m^2^) who were newly diagnosed with type 2 diabetes (intravenous plasma glucose concentration≥11.1mmol/l or fasting blood glucose concentration≥7.0mmol/l) together with 12 control subjects as the control group (age: 26-33 years, BMI: 21-28 kg/m^2^). Fasting blood samples were collected for the measurement of glucose, insulin, 2-hour postprandial blood glucose (PBG2h), and glycated hemoglobin (HbAlc). The magnetic resonance scan of the lower extremity and abdomen was performed, which can evaluate visceral fat content as well as skeletal muscle metabolism and function through transverse relaxation times (T2), fraction anisotropy (FA) and apparent diffusion coefficient (ADC) values.

**Results:**

We found a significant difference in intermuscular fat (IMAT) between the diabetes group and the control group (*p*<0.05), the ratio of IMAT in thigh muscles of diabetes group was higher than that of control group. In the entire cohort, IMAT was positively correlated with HOMA-IR, HbAlc, T2, and FA, and the T2 value was correlated with HOMA-IR, PBG2h and HbAlc (*p*<0.05). There were also significant differences in T2 and FA values between the diabetes group and the control group (*p*<0.05). According to the ROC, assuming 8.85% of IMAT as the cutoff value, the sensitivity and specificity of IMAT were 100% and 83.3%, respectively. Assuming 39.25ms as the cutoff value, the sensitivity and specificity of T2 value were 66.7% and 91.7%, respectively. All the statistical analyses were adjusted for age, BMI and visceral fat content.

**Conclusion:**

Deposition of IMAT in skeletal muscles seems to be an important determinant for IR in type 2 diabetes. The skeletal muscle IMAT value greater than 8.85% and the T2 value greater than 39.25ms are suggestive of IR.

## Introduction

Human organs that can cause insulin resistance (IR) by fat deposition mainly include the heart, liver, skeletal muscles and fat (1) Skeletal muscle fat, which includes subcutaneous adipose tissue (SAT), subfascial adipose tissue (SFAT), intermuscular adipose tissue (IMAT), and intramyocellular lipids (IMCL) (2), is one of the major target tissues of insulin and is important for glucose and lipid uptake and utilization in the human body. Unlike visceral fat, muscle fat and muscle metabolism in relation to the risk of type 2 diabetes has been understudied (3, 4).

Among various fat components of skeletal muscle, IMAT is widely defined as fat infiltration in muscles. Retrospective studies suggest that IMAT may be related to IR and that the increased IMAT may impair muscle blood flow, reduce the ability of insulin to spread, and increase the local concentration of fatty acids (5–7). Under conditions of excess lipid supply, lipid deposition in skeletal muscle is associated with the development of IR, which may even impair insulin signaling (8, 9) However, the previous studies measured the skeletal muscle fat content mainly by DXA, CT or T1-weighted magnetic resonance imaging (T1WI-MRI), which are not accurate enough because of the limited resolution and non-uniformity of the magnetic field (10). The chemical displacement-based water lipid separation (MR Dixon-Quant) is more accurate in measuring fat content (11). Dixon technology is now available for most types of MRI, even for various types of clinical scanning needs (12). In addition, the technology can separate the MRI signals of intramuscular fat, thereby overcome the major limitations of T1WI-MRI (13).

In addition to skeletal muscle fat content, functional MRI indicators also have important value in indicating muscle metabolism. Hence, the relationship between skeletal muscle and magnetic resonance parameters, and their contribution to the risk of type 2 diabetes is important for understanding the pathophysiology of diabetes. Fractional anisotropy (FA) value can detect the water migration rate and limitation in the tissue, and is used to evaluate the microstructure of the tissue (14). Apparent diffusion coefficient (ADC) value can be used to distinguish the difference between normal and pathological skeletal muscles (15). The transverse relaxation times (T2) value can replace the routine laboratory test to quantitatively analyze the skeletal muscle injury (16). However, the associations between the functional MRI muscle indicators (FA, ADC and T2 values) and type 2 diabetes have rarely been studied. The relationships between muscle fat content and functional MRI muscle indicators are also not well understood.

Therefore, we aimed to investigate the association of diabetic biomarker with the skeletal muscle fat content and functional muscle MRI indicators, test the association between muscle fat content and functional MRI muscle indicator, and explore the diagnostic value of skeletal muscle fat content and functional MRI muscle indicators in type 2 diabetes.

## Material and Methods

### Subjects and Experimental Design

A total of 24 male subjects were recruited from December 2017 to December 2018 for this cross-sectional study. Of them, twelve newly diagnosed type 2 diabetic patients (intravenous plasma glucose concentration ≥11.1mmol/l or fasting blood glucose concentration ≥7.0mmol/l) were recruited from the First Endocrinology Clinic of Shengjing Hospital, Shenyang, China. Twelve control young adults were also recruited from the geographic area of Shenyang, China. The inclusion criteria were: aged 25 to 40 years; body mass index (BMI) was between 21 to 29 kg/m^2^; weight was stable within the past 3 months; not following a special exercise program within the past 3 months; non-smoking; no acute illness; no history of diabetes and related family history; no hypertension, cerebrovascular disease, coronary heart disease, chronic heart failure or high uric academia; and have no contraindications for magnetic resonance imaging.

Participants started fasting at 8 pm before the testing day, and had venous blood samples for the measurement of fasting blood glucose, fasting insulin, 2-hour postprandial blood glucose (PBG2h) and glycated hemoglobin (HbAlc) at 7-8 am and 9-10 am at the test of the day. The lower extremity magnetic resonance scans were performed at 12 am. Of notes, for the patients with type 2 diabetes, the magnetic resonance scans of the lower extremity were performed within the first three weeks of diagnosis.

The China Medical University Institutional Review Board approved the study protocols and all subjects provided written and informed consent for their participation. The current study is registered under 2019PS443K.

### Anthropometric Measurements

Subjects’ height ( ± 0.1cm) and weight ( ± 0.1kg) were measured using a wall-mounted stadiometer and a digital balance scale, respectively. These measurements were used to calculate BMI (kg/m^2^).

### Laboratory Assays

All the laboratory assays were processed at the Central Laboratory of Shengjing Hospital of Shenyang, China. Fasting blood glucose and 2-hour postprandial blood glucose were measured within 2 hours after centrifugation (glucose oxidase assay, Olympus 400 automatic biochemical analyzer). HbA1c was detected on the same day by high performance liquid chromatography, Bio Rad D-10, Bole company of the United States. Radioimmunoassay was used to determine fasting plasma insulin (FINS). The homeostatic model assessment of insulin resistance (HOMA-IR) was calculated as: fasting blood glucose × fasting insulin/22.5.

### Magnetic Resonance Imaging and Image Analyses

MRI scans (sagittal T2W1, coronal T2W1, functional imaging T2-mapping, DTI, mDixon-Quant sequences) were performed by Philip Intera Achieva 3.0T scanner to evaluate the cross-sectional areas of the visceral fat of the abdomen (14 cases, 7 newly diagnosed type 2 diabetic patients and 7 controls) and the skeletal muscle fat of the thigh, and the magnetic resonance parameter values (T2 value, FA value, and ADC value) of the skeletal muscle were measured.

**Figure 1:**
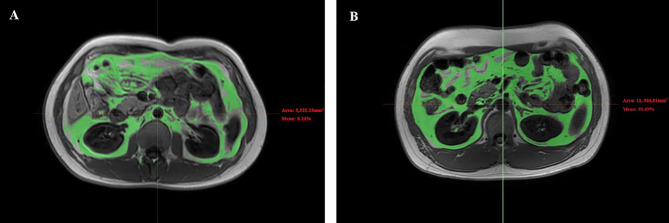
Representative abdomen MRI images of VAT between diabetes group and control group. Representative abdomen MRI images in a healthy volunteer **(A)** and a patient with diabetes **(B)**. Results for the percentage of the VAT were 8.21% for the healthy volunteer and 10.45% for the patient with diabetes. VAT, visceral fat.

Axial images of the mid-L2 vertebral level in MRI data were selected for VAT measurements. The location method was also assisted by the coronal plane in MRI. Then, the relevant single slice Digital Imaging and Communications in Medicine files were imported into Mimics Research software version 21.0 (Materialise, Leuven, Belgium) for image segmentation of VAT (**Figure 1**). The lower extremity scans were obtained at femoral neck, proximal to the terminal end of the femur. This site was chosen because it is the region with the largest skeletal muscle fat, and there is little variability across persons (17). The scan time was approximately 20-25 minutes (**Table 1**). Images of cross-sectional areas of the muscle and fat tissue were analyzed by using Philips Research Imaging Development Environment (PRIDE) software (version 4.1.V3).

**Table 1:** Magnetic resonance sequence acquisition parameters.

Scanning parameter	T1	T2	DTI	T2 mapping	mDIXON-quant
TR	260	5384	3357	2010	9.1
TE	15	100	84	40/60/80/100	1.33
FOV	400	400	410	414	420
SNR	1.00	1.00	1.00	1.00	1.00
NSA	3	2	2	2	2
Thickness/Pitch	4.0/0.4	4.0/0.4	4.0/5.0	4.0/15	6.0/-3.0
Scan time	2:09	2:10	3:13	2:13	0:09

TR, time of repetition; TE, time of echo; FOV, field of view; SNR, signal to noise ratio; NSA, number of signal averaged.

**Figure 2:**
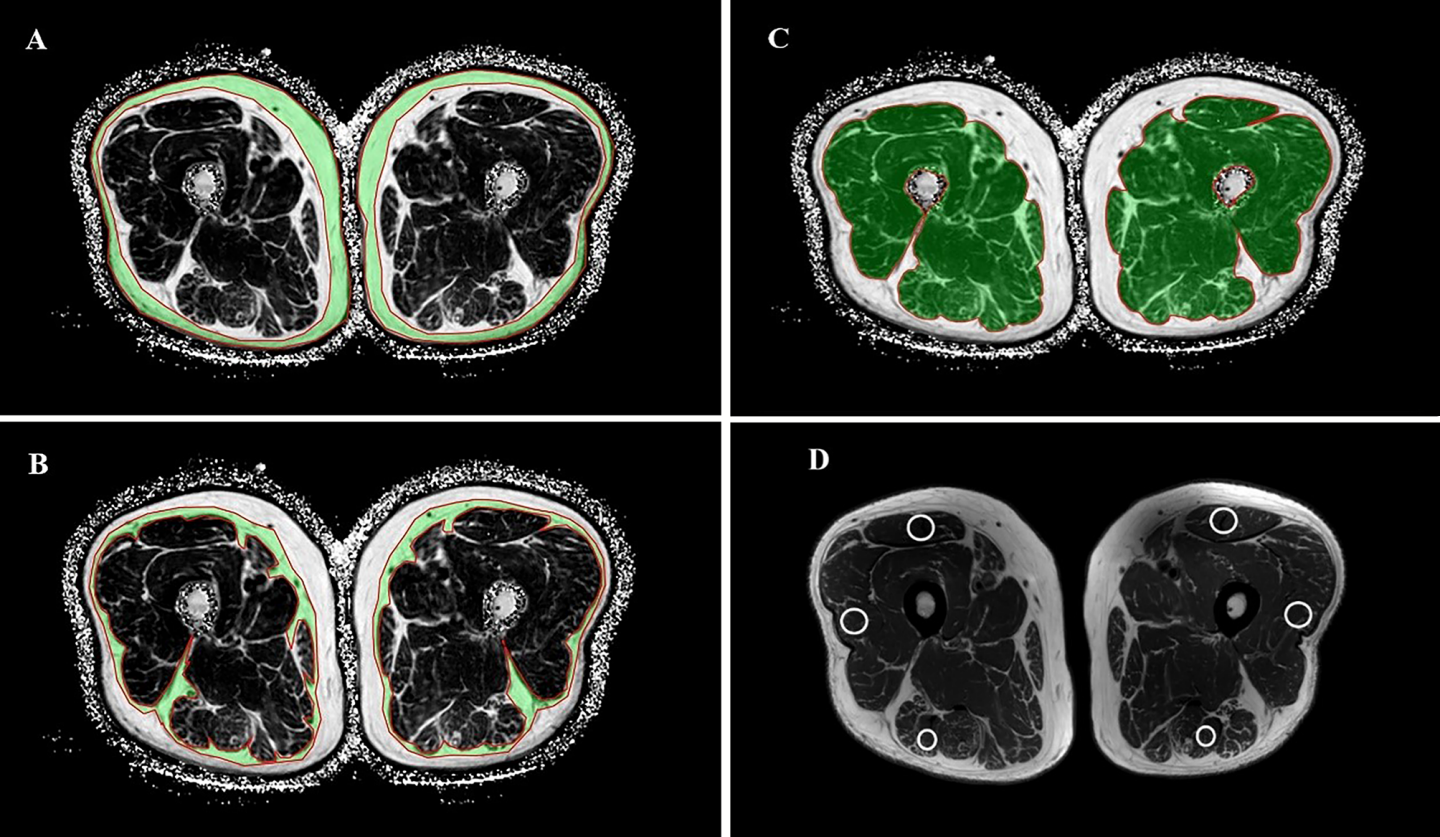
Selection and measurement of mDixon-Quant sequence, T2mapping and DTI sequence ROI. Regions of interest for SAT **(A)**, SFAT **(B)**, IMAT **(C)**, and the positions of the ROI on the rectus femoris, the biceps femoris and the lateral femoral muscle in T2 sequence **(D)**.

By drawing ROI, the thigh MRI image can be segmented into bone and soft tissue measures. For this current study, we measured 1) the percentage of SFAT, IMAT, and SAT in skeletal muscle; 2) T2 value, FA value and ADC value in skeletal muscle (**Figure 2**).

### Statistical Analysis

All data were checked for normality using the Shapiro-Wilk’s test. The measurement data was expressed as mean ± standard deviation (SD). The magnetic resonance parameters and the fat content of the skeletal muscle were compared between the diabetes group and the control group by Binary logistic. Partial variance correlation analysis was carried out to test the associations between magnetic resonance parameters (T2, ADC and FA values) and type 2 diabetes markers (HOMA-IR, PBG2h and HbAlc). Multiple linear regression models were used to assess the association of IMAT and SFAT and SAT with type 2 diabetes markers (HOMA-IR, PBG2h and HbAlc) and magnetic resonance parameters (T2, ADC and FA values). The missing values of the visceral fat content were replaced with the average values in each group. All analyses were adjusted for age, BMI and visceral fat content. The ROC curve was drawn to analyze the value of the above indicators for the diagnosis of type 2 diabetes alone. *p* < 0.05 was considered statistically significant. All analyses were performed using SPSS 23.0 for Windows (SPSS Inc., Chicago, IL, USA).

## Results

### General Characteristics of the Subjects

A total of 24 subjects were included in the study. Subjects in the diabetes group were older, and had higher levels of HOMA-IR, HbA1c, and PBG2h compared to the ones in the control group (*p*<0.05) (**Table 2**).

**Table 2:** General characteristics.

	Diabetes group	Control group	*p*-value
N	12	12	—
Age (years)	34.33 ± 3.98	28.92 ± 3.52	0.002
BMI (kg/m^2^)	26.73 ± 1.98	24.78 ± 3.27	0.091
HOMA-IR	5.74 ± 3.28	1.83 ± 0.75	0.003
HbAlc (mg/dl)	6.67 ± 1.35	5.20 ± 0.21	0.001
PBG2h (mg/dl)	11.11 ± 2.58	4.960.91	<0.001
Visceral fat (%)	8.36 ± 2.37	6.86 ± 3.58	0.238

BMI, body mass index; HOMA-IR, homeostatic model assessment of insulin resistance; HbAlc, glycosylated hemoglobin; PBG2h, 2-hour postprandial blood glucose.

### Comparison of Muscle Magnetic Resonance Parameters and Muscle Fat Components Between Diabetic and Control Subjects

Subjects in the diabetes group had higher levels of IMAT, T2, and ADC compared to the ones in the control group (*p*<0.05) (**Table 3**).

**Table 3:** Comparison of muscle magnetic resonance parameters and muscle fat components in diabetes group (N = 12) and control group (N = 12).

	Diabetes group	Control group	OR	95%Cl	*p*-value
IMAT (%)	10.08 ± 1.37	7.06 ± 2.02	3.27	1.14-9.35	0.03
SFAT (%)	3.69 ± 0.60	3.51 ± 0.79	1.07	0.16-7.44	0.94
SCAT (%)	20.13 ± 6.43	25.19 ± 6.56	0.94	0.77-1.13	0.50
Total fat (%)	33.91 ± 6.35	35.78 ± 7.24	1.02	0.82-1.26	0.86
FA	0.56 ± 0.05	0.51 ± 0.04	2.38	0.89-6.41	0.09
T2 (ms)	40.67 ± 3.75	37.25 ± 1.94	1.75	1.00-3.05	0.05
ADC (10^-3mm^2^/s)	1.20 ± 0.97	0.96 ± 0.26	1.12	1.01-1.23	0.02

All estimates were adjusted for age, BMI and visceral fat content. IMAT, Intermuscular Fat; SFAT, subfascial adipose tissue; SAT, subcutaneous adipose tissue; T2, transverse relaxation times; FA, fractional anisotropy; ADC, apparent diffusion coefficient; OR, odds ratio; Cl, confidence interval.

### Correlation Between Muscle Magnetic Resonance Parameters and Type 2 Diabetes Markers

Among the three selected magnetic resonance parameters (T2, FA, and ADC), muscle T2 value ​​and FA value ​​were associated with HOMA-IR, PBG2h and HbAlc (*p*<0.05). ADC value was associated with PBG2h (*p*<0.05) (**Table 4**).

**Table 4:** Correlation between muscle magnetic resonance parameters and type 2 diabetes markers (N = 24).

	HOMA-IR	HbAlc (mg/dl)	PBG2h (mg/dl)
	*p*-value	*p*-value	*p*-value
T2 (ms)	<0.001	<0.001	0.020
ADC (10^-3mm^2^/s)	0.071	0.052	0.045
FA	0.005	0.001	0.001

All estimates were adjusted for age, BMI and visceral fat content. T2, transverse relaxation times; FA, fractional anisotropy; ADC, apparent diffusion coefficient; HOMA-IR, homeostatic model assessment of insulin resistance; HbAlc, glycosylated hemoglobin; PBG2h, 2-hour postprandial blood glucose.

### Associations Between Thigh Fat Composition and Muscle Magnetic Resonance Parameters and Type 2 Diabetes Markers

In the models including IMAT, SFAT and SAT, IMAT was significantly associated with type 2 diabetes markers (HOMA-IR, PBG2h and HbAlc) and magnetic resonance parameters (T2, ADC and FA values) (*p*<0.05), and SFAT was significantly associated with HOMA-IR (*p*<0.05). However, SAT was not associated with type 2 diabetes markers (HOMA-IR, PBG2h and HbAlc) and magnetic resonance parameters (T2, ADC and FA values) (*p*>0.05) (**Table 5**).

**Table 5:** Association between thigh fat composition and muscle magnetic resonance parameters and type 2 diabetes markers (N = 24).

Model		HOMA-1R		HbAlc (mg/dl)		PBG2h (mg/dl)		T2 (ms)		FA	
		β	*p*-value	β	*p*-value	β	*p*-value	β	*p*-value	β	*p*-value
1	IMAT	1.025	<0.001	0.587	<0.001	1.190	0.001	1.464	<0.001	0.019	0.002
2	SFAT	1.082	0.018	0.441	0.275	1.100	0.416	2.207	0.123	0.027	0.216
3	SAT	-0.087	0.060	-0.096	0.058	-0.232	0.070	-0.335	0.244	-0.002	0.391
4	IMAT	0.511	<0.001	0.614	<0.001	1.213	0.004	1.390	0.001	0.018	0.008
	SFAT	0.527	0.046	-0.255	0.454	-0.216	0.849	0.699	0.520	0.008	0.680
5	IMAT	0.573	<0.001	0.581	<0.001	1.096	0.010	1.607	<0.001	0.021	0.005
	SAT	0.004	0.891	-0.004	0.924	-0.058	0.629	0.089	0.444	0.001	0.487
6	IMAT	0.485	<0.001	0.623	<0.001	1.106	0.020	1.528	0.002	0.020	0.014
	SFAT	0.563	0.047	-0.269	0.465	-0.063	0.958	0.501	0.664	0.001	0.827
	SAT	-0.013	0.640	0.005	0.901	-0.056	0.665	0.073	0.554	0.005	0.559

Multivariate linear regression analysis was used; All estimates were adjusted for age, BMI and visceral fat content. Type 2 diabetes markers (HOMA-IR, PBG2h and HbAlc) and magnetic resonance parameters (T2, ADC and FA values) were dependent variables. IMAT, SFAT and SAT were independent variables of model 1-3, respectively. The independent variables of model 4-5 were paired by IMAT, SFAT, and SAT. The independent variables of model 6 included IMAT, SFAT and SAT. IMAT, intermuscular adipose tissue; SFAT, subfascial adipose tissue; SAT, subcutaneous adipose tissue; HOMA-IR, homeostatic model assessment of insulin resistance; HbAlc, glycosylated hemoglobin; PBG2h, 2-hour postprandial blood glucose; T2, transverse relaxation times; FA, fractional anisotropy.

### The Diagnostic Value of IMAT, FA, and T2 Values for Type 2 Diabetes

**Figure 3:**
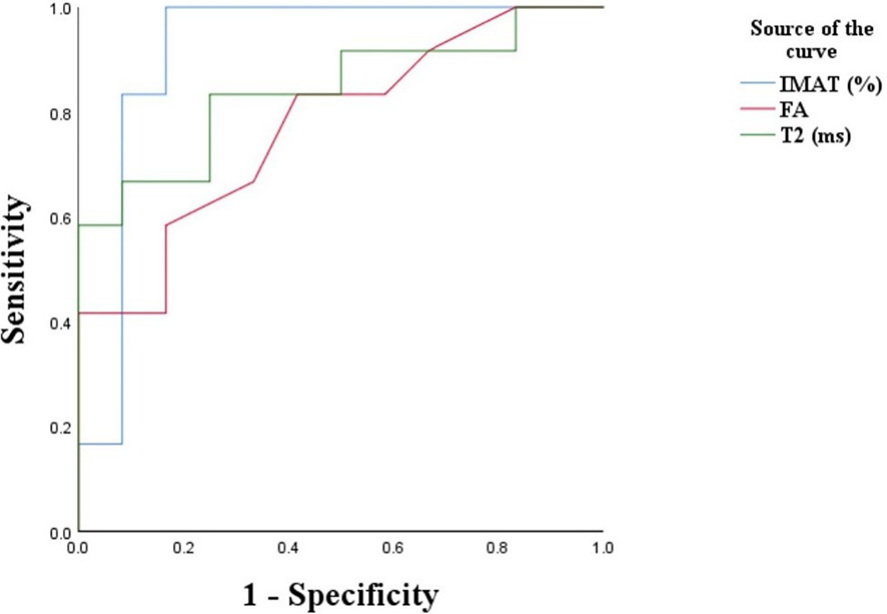
ROC plot of each type of indicator for diagnosis of type 2 diabetes. IMAT, intermuscular adipose tissue; FA, fractional anisotropy; T2, transverse relaxation times.

**Figure 2** presents the ROC curve of type 2 diabetes based on T2, FA and IMAT, which showed significant associations with HOMA-IR, PBG2h and HbAlc values of type 2 diabetes. The selection of the cutoff value was based on the maximization of the Youden’s index. The cut-off value of IMAT was 8.85%, its sensitivity to type 2 diabetes was 100%, specificity was 83.3%. The cutoff value of T2 value was 39.25ms, and its diagnostic sensitivity was 66.7% and specificity was 91.7%. The cutoff value of FA value was 0.58, and its diagnostic sensitivity was 41.7% and specificity was 91.8%. The area under the ROC (AUROC) for the diagnosis of type 2 diabetes by IMAT, T2 and FA values ​​were 0.917, 0.840 and 0.774, respectively. Among these indicators, IMAT appeared to be the best predictor, followed by T2 and FA (**Figure 3**).

## Discussion

In this study, we show that IMAT is associated with IR among young adults, independent of age, BMI and visceral fat content. However, SAT, which makes up the highest proportion of muscle fat, is not associated with IR. The T2 and FA values are also positively correlated with IR, which provides new evidence for the relationship between the muscle magnetic resonance index and IR. In addition, IMAT discriminates type 2 diabetic patients from control subjects with a sensitivity of 100%, and a specificity of 83.3%.

The mechanism underlying the relationship between skeletal muscle fat and IR is still not clear. Previous studies have shown that IMAT may be related to multiple metabolic factors (18–21), this association is often attributed to the close relationship between IMAT and BMI, Some studies also suggested that due to the adjacent anatomical structure of skeletal muscle and IMAT, IMAT may affect the direct contact between fat and muscle cells through the secreted adipokines or locally affect the muscles through the shared micro-vessels (22, 23). The infiltration of IMAT in skeletal muscle may also increase the local inflammation of muscle fibers, which may lead to increased oxidative stress in the muscles. This could result in decreased insulin-stimulated tyrosine phosphorylation and decreased activity of downstream signaling molecules, which in turn causes insulin resistance (24, 25). Recent research indicates that IMAT may regulate muscle insulin sensitivity by secreting inflammatory cytokines and extracellular matrix proteins, as well as increasing the concentration of free fatty acids (FFA) in the body (26). Our study found that IMAT was statistically different between type 2 diabetic patients and control subjects. There was no significant difference in SFAT or SAT between the two groups. Moreover, we showed that IMAT was associated with higher HOMA-IR, HbA1c, and PBG2h. Our findings are consistent with the results of Goodpaster et al. (18, 27). They evaluated the adipose tissue of thighs in 65 Americans by CT and DXA and suggested that IMAT and SFAT might be markers of IR in type 2 diabetes. Another study found that high levels of thigh subcutaneous adipose tissue and low levels of thigh IMAT might maintain good insulin metabolism in early postmenopausal American women (19). In addition, Kim et al. (20) reported that among 75 middle-aged and older adults, IMAT might have a negative influence on fasting glucose concentration. These findings collectively indicate that IMAT may increase metabolic risk in IR and type 2 diabetes. In summary, we speculate that IMAT may increase in the skeletal muscle of Chinese male patients with type 2 diabetes, which could affect the muscle mass and muscle function.

There are a few studies regarding the correlation between skeletal muscle MR parameters and IR, as well as type 2 diabetes (28, 29), and whether it can be used as an index to predict or diagnoses type 2 diabetes still remains unclear. Their findings indicate that fasting hyperinsulinemia (insulin resistance) and dyslipidemia have independent and additional contributions to increased tissue magnetic resonance T2 values, and T2 values should be early screened for metabolic dysfunction to prevent diabetes and cardiovascular disease (30). Others have shown that biomarkers associated with water T2 can provide evidence for the pathophysiology of metabolic syndrome and early metabolic disorders prior to the occurrence of type 2 diabetes and cardiovascular disease (31). DTI sequence and its parameters (ADC and FA values) can be used to evaluate the difference of fibrous tissue in diseased and control muscle tissues, and as one of the commonly used methods. It can also provide an in-depth understanding of muscle dynamics, which may apply the analysis and research of muscle tissue metabolism (32, 33). Among muscle fat components, IMAT has a positive correlation with T2 and FA values, which indicates that IMAT may affect muscle metabolism and participate in the process of early IR in type 2 diabetes.

In recent years, various types of diabetes prediction models have been proposed (34–37), but none of those have been constructed or validated in skeletal muscle fat content or magnetic resonance parameters in the Chinese population. Moreover, our model was based on MR mDIXON-quant quantitative technique, and IMAT and T2 value that may affect the incidence of the disease were included in the regression model. Our results showed that among the equal magnetic resonance parameters, T2 and FA values were associated with increased HOMA-IR and HbA1c. It is indicated that T2 can detect abnormalities in skeletal muscle signals in newly diagnosed diabetic patients. IMAT discriminated type 2 diabetic patients from control subjects with the sensitivity of 100% and the specificity of 83.3% when setting the cutoff value of IMAT as 8.85%. Muscle T2 value might also be used to detect early skeletal muscle changes in type 2 diabetes patients, assuming 39.25ms as the cutoff value, the sensitivity and specificity were 66.7% and 91.7% respectively. In our study, the IMAT and SFAT levels of one control subject were higher than the cut-off value, but the HOMA-IR, PBG2h and HbAlc values were normal. During 2-year follow up period, his fasting insulin and fasting blood glucose were found to have exceeded the normal value. After clinical consultation, treatment options including medication and exercise therapy were developed for this subject.

At current stage, various weight loss guidelines rarely consider specific organs (e.g., for skeletal muscle and pancreas) (38, 39). MR imaging provides a unique, non-radiative and non-invasive diagnostic platform that can directly quantify the physiological and biochemical variables of skeletal muscle (40). Using more accurate MR analysis to analyze the content of IMAT and the measurement of muscle magnetic resonance signals may provide clinicians with more specific strategies for treating skeletal muscle fat infiltration, and even predict the long-term risk of type 2 diabetes in obese patients.

The employment of MR mDIXON-quant quantitative technique to quantify IMAT is a strength of this study. This technique is able to directly measure IMAT and more sensitive than computed tomography (CT), which indirectly measures IMAT (41). Type 2 diabetes has many confounding factors such as medications, but the patients in our study were all newly diagnosed with type 2 diabetes and not on medications, which is another strength.

There are several limitations in our study. First, our sample size is modest, and we did not calculate our sample size before conducting this study. However, as a *post hoc* analysis, we found that the average IMAT was 10.08% with an SD of 1.37 in the diabetes group, and the average was 7.06% with an SD of 2.02 in the control group. Based on our data, this study had a power of 98.24% to detect the difference in IMAT between the diabetes group and the control group at 5% significance level with a total of 24 samples equally allocated between the two groups. In other words, despite the modest sample size, we were still able to identify the significant differences between the two groups. Second, this study is cross-sectional, and no causal relationship can be derived. Prospective follow-up studies with an adequate sample size are needed to validate our findings. Third, because the mDIXON-quant technique scan image used was susceptible to motion artifacts, and image post-processing only by manually delineating ROI to measure the content of various fat components in muscles, there could be measurement errors. Fourth, the missing values of the visceral fat content, when used as a confounding factor in the statistical analyses, might cause biased results. Fifth, our participants are all Chinese males, such that the findings cannot be generalized to other populations. Last, MRI scanning is costly, and not widely used yet. To the best of our knowledge, the relationship between the fat components of muscles and the prevalence of type 2 diabetes is understudied (42). Thus, at this stage, mDIXON-Quant, a robust noninvasive quantitative method available, would be well suited for the accurate measurement of the content of IMAT and muscle magnetic resonance signals in this regard.

In conclusion, IMAT in skeletal muscles is associated with IR, and T2 value can detect the metabolic irregularity of skeletal muscle in Chinese male patients with newly diagnosed type 2 diabetes. The skeletal muscle IMAT value greater than 8.85% and the T2 value greater than 39.25ms are suggestive of type 2 diabetes in Chinese males.

## Data Availability Statement

All datasets generated for this study are included in the article/supplementary material.

## Ethics Statement

The studies involving human participants were reviewed and approved by 2019PS443K. The patients/participants provided their written informed consent to participate in this study. Written informed consent was obtained from the individual(s) for the publication of any potentially identifiable images or data included in this article.

## Author Contributions

FY, SP, and HZ conceived and designed research. FY performed experiments, analyzed data and drafted manuscript. BH and FW provided clinical support for the experiment. LC, HZ, and YD edited and revised manuscript. SP approved the final version of manuscript. All authors contributed to the article and approved the submitted version.

## Funding

This work was supported by 345 Talent Project and Natural Science Foundation of Liaoning Province grant number [No.2019-ZD-0794].

## Conflict of Interest

The authors declare that the research was conducted in the absence of any commercial or financial relationships that could be construed as a potential conflict of interest.
